# Prescribing Controversies: An Updated Review and Meta-Analysis on Combined/Alternating Use of Ibuprofen and Paracetamol in Febrile Children

**DOI:** 10.3389/fped.2019.00217

**Published:** 2019-06-05

**Authors:** Giulia Trippella, Martina Ciarcià, Maurizio de Martino, Elena Chiappini

**Affiliations:** ^1^Department of Health Sciences, Post-Graduate School of Pediatrics, Anna Meyer Children's University Hospital, University of Florence, Florence, Italy; ^2^Department of Health Sciences, University of Florence, Florence, Italy; ^3^Division of Pediatric Infectious Disease, Department of Health Sciences, Anna Meyer Children's University Hospital, University of Florence, Florence, Italy

**Keywords:** children, antipyretics, fever, ibuprofen, paracetamol, acetaminophen

## Abstract

**Background:** Ibuprofen and paracetamol are the only antipyretics recommended in febrile children. According to international guidelines the choice of the drug should rely on the child's individual characteristics, while a controversial issue regards the combined or alternate use of the two drugs.

**Objective:** To compare the efficacy and safety of combined or alternating use of ibuprofen and paracetamol in children.

**Methods:** A systematic review of literature was performed on Medline and Embase databases. The included studies were randomized controlled trials analyzing the efficacy of combined or alternating therapy with antipyretics in febrile children vs. monotherapy. A meta-analysis was performed to measure the effect of treatment on child's temperature and discomfort. Adverse effects were analyzed as secondary outcome.

**Results:** Nine studies were included, involving 2,026 children. Mean temperature was lower in the combined therapy group at 1 h (mean difference: −0.29°C; 95%CI: −0.45 to −0.13) after the initial administration of therapy. No statistical difference was found in mean temperature at 4 and 6 h from baseline. A significant difference was found in the proportion of children reaching apyrexia at 4 and 6 h with the combined treatment (RR: 0.18, 95%CI: 0.06 to 0.53, and 0.10, 95%CI: 0.01–0.71, respectively) and at 6 h with alternating treatment (RR: 0.30, 95% CI: 0.15–0.57), compared to children treated with monotherapy. The child's discomfort score was slightly lower with alternating therapy vs. monotherapy. The pooled mean difference in the number of medication doses per child used during the first 24 h was not significantly different among groups.

**Discussion:** Combined or alternating therapy resulted more effective than monotherapy in reducing body temperature. However, the benefit appeared modest and probably not clinically relevant. The effect on child discomfort and number of doses of medication was modest as well. According to our findings, evidences are not robust enough to encourage combined or alternating paracetamol and ibuprofen instead of monotherapy to treat febrile children, reinforcing the current recommendation of most of the international guidelines.

## Introduction

Fever is a physiologic host defense mechanism to infections. It is one of the most common symptoms in children and it is usually associated with a self-limiting viral infection (1). However, it leads to frequent concerns for parents and caregivers, which may result in “fever phobia,” over-treatment and incorrect management of the febrile child (2–5). Most experts support the use of antipyretics in febrile children with the main purpose to alleviate the child's discomfort, when present, rather than to achieve normothermia (6).

Ibuprofen and paracetamol are the only antipyretics recommended in febrile children, basing on abundant evidence of their efficacy and safety profile. A recent systematic literature review (7), including eight studies on 1,632 children, concluded that both drugs are equally effective in reducing fever and discomfort in children. Other literature reports, including two meta-analyses (8, 9), observed a similar safety profile (10). In a meta-analysis of 19 studies, no significant difference was observed between the two drugs in the incidence of adverse events in febrile children (0.82; 95% CI 0.60–1.12) (9).

According to guidelines recommendations, the choice of the drug should rely on the child's individual characteristics, such as age, underlying clinical conditions, co-medications (11).

A controversial issue regards the combined or alternate use of the two drugs. Combined therapy is defined as the simultaneous administration of the two drugs, while alternating therapy is the administration of both drugs at different time points. Epidemiological studies reported that these practices are widespread among pediatricians, nurses, pharmacists, and caregivers, in different countries. In a recent Italian survey, 12% of 800 interviewed pediatricians declared combined or alternating prescription habit (12). Higher proportions have been reported in Switzerland (65%), USA (50%), Turkey (91%), and Spain (69%) (13–15).

However, there is uncertainty about whether combined/alternate therapy is superior to the single drug therapy, especially in terms of discomfort reduction and about adverse effect profile. Potential renal and liver toxicity caused by the additive effects of drug metabolites in children has been suggested. Moreover, some authors underlined the possibility that caregivers will either not receive or not understand dosing instructions, increasing the potential for inaccurate dosing or overdosing. Finally, it has been argued that the combination strategy is mainly driven by the healthcare professionals' and caregivers' fever phobia and it could be limited with targeted educational interventions (16, 17).

According to a French cross-sectional observational study (18), combination treatment was associated with: parents' profession being a farmer, possibly related to the difficulty in implementing recommendations in rural areas; a diagnosis of particularly painful (otitis, pharyngitis) and feverish diseases (influenza); child's temperature major than 39°C, probably due to the persistence of fever phobia. Furthermore, the study revealed that parents' knowledge and practices in managing fever symptoms frequently differ from recommendations (18, 19).

The rational for the combined or alternate use of ibuprofen and paracetamol is that a synergism between the two drugs is plausible, given their different mechanisms of action (17). The combination of paracetamol and ibuprofen has been found to be effective in a variety of acute pain states, including post-operative pain, dysmenorrhea, musculoskeletal pain, and tooth removal associated pain (17). This is particularly important considering that discomfort and pain relief is the main objective of fever treatment. Several clinical trials and systematic reviews have been published with the aim to explore the effect of combined/alternate therapy on temperature control and child's discomfort, but results are contrasting (20, 21).

A 2013 Cochrane Review of six studies, involving 915 participants, concluded that some evidence exists that combined or alternate antipyretic therapy may be slightly superior at reducing temperatures than monotherapy. Nonetheless, evidence for improvements in child's discomfort, which should be the primary aim of treatment, were poor. Moreover, the authors identified concerns regarding the safety of combined and alternating therapy: even if no serious adverse events were documented in the trials, no study had sufficient power in terms of number of participants to make a definitive statement about frequency of severe adverse effects (22).

Currently the alternate use of two antipyretics is discouraged by several guidelines with the exception of the UK-NICE (23) and South Australian CPGs (24). These two guidelines permit the alternate use only if the discomfort persists or recurs after the administration of one antipyretic.

On the other hand, the Canadian (25), Italian (11), New Zealand (26) and South African (27) guidelines discourage this practice in every case, while the US guidelines (28) state that there is insufficient evidence to support or refute the routine use of combined/alternating treatment and “practitioners who follow this practice should counsel parents carefully regarding proper formulation, dosing and dosing intervals, and emphasize the child's comfort instead of reduction of fever” (29). The current international guidelines are summarized in Table 1 (30).

**Table 1:** Management of fever guidelines (from 29, modified).

**Guidelines**	**AAP**	**SIP**	**South- Africa**	**NICE**	**NSW**	**SA**	**WHO**	**CANADA**
Country	USA	Italy	South Africa	UK	New Zealand	South Australian		Canada
Year	2011	2016	2013	2013	2010	2013	2013	2013
Age of target population	Not specified	0–18 years	Not specified	<5 years	1 month−5 years	<3 years	<5 years	Not-specified
**SPECIFIC RECOMMENDATIONS**								
Combination of paracetamol/ibuprofen is not recommended	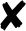 (1)	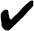	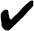	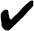	nr	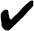	nr	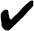 (3)
Alternating paracetamol/ibuprofen is not recommended	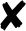 (1)	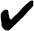	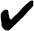	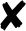 (2)	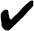	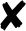 (2)	nr	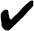 (3)

*
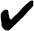
Agree*.

*
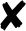
Disagree*.

*nr, not reported*.

*(1) Insufficient evidence to support or refute the routine use of combination treatment*.

*(2) Alternating the two drugs is possible if the discomfort persists or recurs using only one antipyretic*.

*(3) Do not alternate between using acetaminophen and ibuprofen as this can lead to dosing errors*.

Hereby, we perform an updated systematic review and meta-analysis regarding the efficacy and safety of combined or alternate use of antipyretics in children, in order to provide a state-of-the art summary, 5 year after the 2013 Cochrane review (22).

## Materials and Methods

### Study Design

A systematic review was performed according to the Cochrane handbook guidelines (31). The aim of the review was to assess the efficacy and safety of combined or alternating use of ibuprofen and paracetamol vs. monotherapy in febrile children. The included studies were evaluated qualitatively and quantitatively with a meta-analysis. The PRISMA guideline recommendations were used to report this systematic review and meta-analyses (32).

### Search Strategy

On September 2018, the MEDLINE and EMBASE databases were searched.

In MEDLINE the following search strategy was used: (ibuprofen AND (fever OR pyrexia OR hyperthermia OR temperature OR febrile OR feverish) AND (infan^*^ OR child^*^ OR pediatric^*^ OR pediatric^*^ OR adolescen^*^).

In EMBASE the used search strategy was: “ibuprofen”:ab,ti AND (“fever”:ab,ti OR “pyrexia”:ab,ti OR “hyperthermia”:ab,ti OR “temperature”:ab,ti OR “febrile”:ab,ti OR “feverish”:ab,ti) AND (“infan^*^”:ab,ti OR “child^*^”:ab,ti OR “pediatric^*^”:ab,ti OR “pediatric^*^”:ab,ti OR “adolescen^*^”:ab,ti) AND AND (“clinical trial”/de OR “controlled clinical trial”/de OR “prospective study”/de OR “randomized controlled trial”/de OR “randomized controlled trial (topic)”/de).

An additional research was conducted on Google Scholar and the references of relevant articles were further crosschecked. No language restriction or publication date restrictions were applied.

### Inclusion Criteria

Studies were considered eligible whether they presented the following characteristics: (1) study design consisting in randomized controlled trial (RCT), open or blinded; (2) population sample represented by children aged 18 or less; (3) one of the arms of therapy consisting of combined or alternating antipyretics for fever treatment; (4) available data to measure the effect of therapy.

### Selection of Studies

Titles and abstracts were screened to identify eligible studies. Then, the full text of all potentially relevant articles was analyzed for further evaluation. Two investigators (GT and EC) independently reviewed and evaluated every study. Disagreements were resolved by consensus.

### Data Extraction and Definitions

The following information was extracted: publication year, country of origin, characteristics of population sample, diagnostic measures of fever, types of intervention, outcome measures.

Types of interventions were: combined therapy vs. ibuprofen or paracetamol alone, alternating therapy vs. ibuprofen or paracetamol alone, or combined therapy vs. alternating therapy.

The primary outcome measures were: mean temperature and proportion of febrile children at one, 4 and 6 h after administration of initial antipyretic, child discomfort evaluated by stress scores (the Non-communicating Children's Pain Checklist, NCCPC) and number of doses of medication given. The NCCPC score is a validate measure of pain and discomfort in children who are unable to speak about their pain (33). Adverse effects of treatments were analyzed as secondary outcome.

Regarding mean reduction in body temperature, there is no international consensus on the definition of a clinically significant reduction. Some authors have stated that only differences of at least 1°C could be considered meaningful (34–36).

Data extraction from the selected articles were carried out independently by two investigators (GT and EC).

### Risk of Bias Assessment

The risk of bias for the studies were assessed using the Cochrane Risk of Bias tool, which is a domain-based evaluation, consisting in six domains: sequence generation, allocation concealment, blinding, missing outcome data, selective outcome reporting, and other sources of bias. Two other potential sources of bias were assessed: the potential influence of funding agencies and relevant disproportions in baseline characteristics among groups (31).

### Statistical Analysis

Meta-analyses were performed for studies reporting similar outcome measures, labeling data by comparisons (combined therapy vs. single agent, alternating therapy vs. single agent, combined therapy vs. alternating therapy).

For continuous outcomes, the mean, and standard deviation of each group were extracted. Then, data were analyzed using mean differences (MD) and 95% confidence intervals (CI). For dichotomous outcomes, the number of events and the number of patients analyzed for each group were extracted. Risk ratio (RR) was used to report pooled results, with 95% CI.

The magnitude of heterogeneity between studies was expressed by the I^2^ index. I^2^ values of 50% were considered to represent moderate heterogeneity, I^2^ values between 50 and 75% substantial heterogeneity and >75% considerable heterogeneity. The Chi2 test was also used to evaluate heterogeneity, with a *P* < 0.1 to represent significant heterogeneity (37).

Estimates of effect were combined using a random-effects model when moderate heterogeneity existed and a fixed-effect model when there was no heterogeneity.

Due to the small number of studies included in the meta-analyses, data were not enough to build funnel plots. Hence, publication bias was not tested.

The statistical analyses were performed using Review Manager 5 (38).

### Levels of Evidence Assessment

The Grading of Recommendation, Assessment, Development, and Evaluation (GRADE) Working Group recommendations (39) were followed to assess the quality of evidence, using the GRADEpro GDT (Guideline Development Tool) (40).

## Results

### Literature Screening

**Figure 1:**
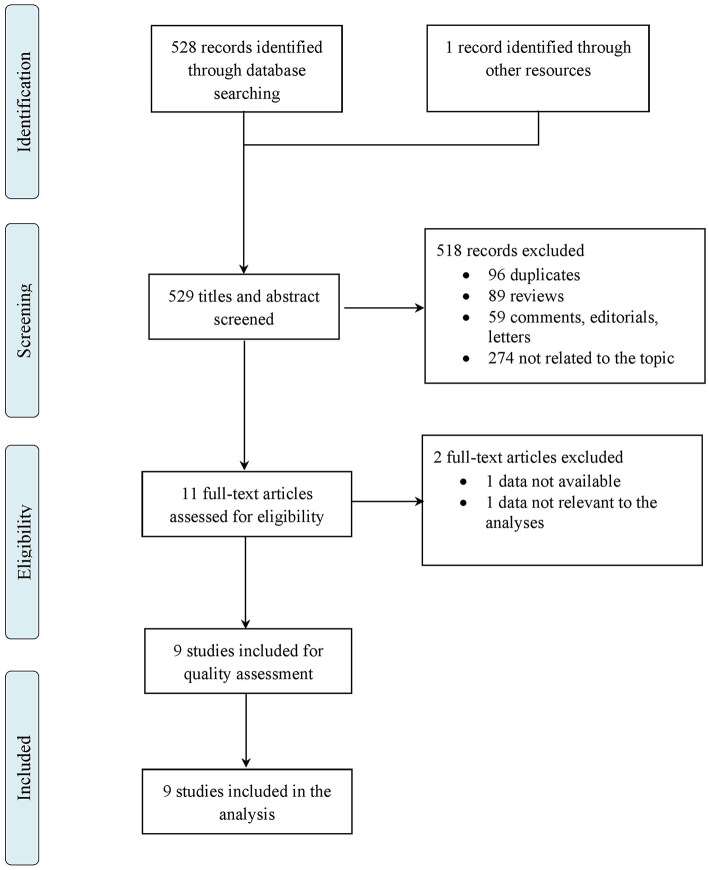
Flow chart of study selection.

The initial search identified 528 records, 393 from MEDLINE database and 135 from EMBASE database, of which 96 were duplicates. One additional record was identified using Google Scholar as search engine. Eleven studies met inclusion criteria after abstracts and titles were evaluated. Two of these studies were excluded because data were not reported or not relevant to the analyses. Nine randomized trial were judged eligible and included in the analysis. The search results and the selection process are shown in Figure 1 (34–36).

### Study Characteristics

This review included three additional studies (36, 41, 42) compared to the 2013 Cochrane review. The included studies were nine RCTs, open or blinded. Patients were a total of 2,026 children, aged 6 months to 14 years, presenting with fever. Settings were emergency departments, pediatric departments or outpatient clinics and services.

The majority of studies defined fever as a body temperature ≥38°C. Only one study considered children with temperature ≥37.8°C as febrile (43).

For temperature measurement, two studies used axillary thermometers (41, 43), two studies used tympanometric thermometers (34, 36), one study used temporal artery thermometers (44), two studies used rectal thermometers (45, 46), and one study used oral thermometers with children >2 years old and rectal thermometers with children <2 years (47). One study did not mention the method used to assess body temperature (42).

In all studies, therapy was administered orally. Seven studies used paracetamol at dose of 15 mg/kg (34, 36, 42–45, 47), one study at a dose of 10 mg/kg (41) and one study at a dose of 12.5 mg/kg (46). Seven studies used ibuprofen at a dose of 10 mg/kg (36, 41–45, 47), two studies at a dose of 5 mg/kg (34, 46). In one study, half of every group received initial loading with paracetamol [25 mg/kg] and the other half received initial loading with ibuprofen (10 mg/kg) (46).

Four studies compared combined therapy vs. ibuprofen and paracetamol alone (34, 36, 42, 43), two studies compared alternating therapy vs. ibuprofen and paracetamol alone (41, 46), one study compared alternating therapy vs. ibuprofen alone (45), one study compared alternating therapy vs. paracetamol alone (47), one study compared combined and alternating therapy vs. ibuprofen alone and alternating vs. combined therapy (44).

Primary outcome measures included: child's body temperature, proportion of children with refractory fever, scores of child discomfort and amount of antipyretic used at different time points. Other outcome measures were: time spent in department, number of emergency department visits, absence from daycare, recurrence of fever, symptom checklist, parental perception of efficacy, incidence of adverse effects.

Study characteristics are summarized in Table 2.

**Table 2:** Characteristics of the included studies.

**References**	**Country/setting**	**Patients**	**Diagnosis of fever**	**Intervention**	**Outcomes**
Erlewyn-Lajeunesse et al. (34)	UK/single center – ED	123 children aged 6 months to 10 years with temperature > 38°C	tympanometric thermometer	Group 1: paracetamol 15 mg/kg single dose Group 2: ibuprofen 5 mg/kg single dose Group 3: paracetamol 15 mg/kg + ibuprofen 5 mg/kg single dose of each	Primary 1. Temperature at 1 h Secondary 1. Temperature at 2 h 2. Time spent in department
Hay et al. (43)	UK/multi-center-−35 primary care sites and households	156 children aged 6 months to 6 years with temperature between 37.8°C and up to 41°C	axillary thermometer	Group A: paracetamol 15 mg/kg every 4–6 h Group B: ibuprofen 10 mg/kg every 6–8 h Group C: paracetamol + ibuprofen	Primary 1. Min without fever (<37.2°C) in the first 4 h 2. Proportion of children normal on the discomfort scale at 48 h Secondary 1. Time to fever clearance in the first 24 h 2. Time spent without fever over 24 h 3. Proportion of children without fever associated symptoms: discomfort, reduced activity, reduced appetite and disturbed sleep at 48 h and day 5 4. Adverse effects
Kramer et al. (47)	USA/single center—pediatric clinic	38 children aged 6 months to 6 years with temperature > 38°C	children > 2 years oral, children <2 years rectal thermometer	Group A: paracetamol (15 mg/kg) alternated with placebo Group B: paracetamol (15 mg/kg) alternating with Ibuprofen (10 mg/kg)	Primary 1. Temperature at enrolment and• h 3, 4, 5, 6 Secondary 1. Symptom checklist at h 3 and 4 2. Parental perception of efficacy at h 3 and 4
Luo et al. (41)	China/single center—ED and pediatric department	474 children aged 6 months to 5 years with temperature > 38.5 C	axillary thermometer	Group 1: alternating acetaminophen 10 mg/kg per dose and ibuprofen 10 mg/kg per dose (acetaminophen with shortest interval of 4 h and ibuprofen with shortest interval of 6 h and the shortest interval between acetaminophen and ibuprofen of 2 h) Group 2: acetaminophen 10 mg/kg per dose with shortest interval of 4 h Group 3: ibuprofen 10 mg/kg per dose with shortest interval of 6 h	Primary 1. NCCPC scores throughout 24 h 2. Mean temperature throughout 24 h Secondary 1. Proportion of children with refractory fever for 4 h 2. Low body temperature 3. Antipyretic use 4. Incidence of adverse events
Nabulsi et al. (45)	Lebanon/multi-center—pediatric inpatient services	70 children aged 6 months to 14 years with temperature > 38.8°C	rectal thermometer	Control: ibuprofen 10 mg/kg followed by placebo 4 h later Treatment group: single oral dose ibuprofen 10 mg/kg followed by single oral dose paracetamol 15 mg/kg 4 h later	Primary 1. Proportion of children with normal body temperature at 6 h Secondary 1. Proportions of afebrile children at 7 and 8 h
Noori et al. (42)	Iran/single center – ED	540 children aged 6 months to 10 years terwith temperature between 38°C and 41°C	not specified	Group 1: acetaminophen 15 mg/kg/dose Group 2: ibuprofen 10 mg/kg/dose Group 3: a combination of acetaminophen plus ibuprofen	Primary 1. Temperature at 2–4–6 h
Paul et al. (44)	USA/single center—outpatient clinics and child day-care facilities	46 children aged 6 months to 8 years with temperature > 38.8°C	temporal artery thermometer	Group A: single dose ibuprofen 10 mg/kg (oral suspension 100 mg/5 mL) Group B: single dose acetaminophen 15 mg/kg (oral solution 160 mg/5 mL) plus ibuprofen 10 mg/kg Group C: ibuprofen 10 mg/kg at the beginning of the study followed by 15 mg/kg of acetaminophen 3 h later	Primary 1. Effect of treatment on temperature over 6 h
Sarrell et al. (46)	Central Israel/multi-center—primary pediatric community ambulatory centers	480 children aged 6 to 36 months with temperature > 38.4°C	rectal thermometer	Group 1: paracetamol 12.5 mg/kg every 6 h Group 2: ibuprofen 5 mg/kg every 8 h Group 3: paracetamol 12.5 mg/kg/dose alternating with ibuprofen 5 mg/kg/dose every 4 h Half of every groups received initial loading with paracetamol (25 mg/kg) and the other half received initial loading with ibuprofen (10 mg/kg)	Primary 1. Body temperature 2. Stress score 3. Amount of antipyretic used at the 3 day time point Secondary 1. Total days that a primary caretaker had to stay home from work 2. Recurrence of fever (≥37.8°C) within 5 and 10 days after initiation of treatment 3. Number of emergency department visits within 10 days of enrolment 4. Hepatic and renal function 5. Appearance of gastrointestinal symptoms or bleeding
Vyas et al. (36)	India/single center—pediatric department	99 children aged 6 months to 12 years with temperature > 38°C	tympanometric thermometer	Group 1: ibuprofen 10 mg/kg single dose Group 2: paracetamol 15 mg/kg single dose Group 3: paracetamol 15 mg/kg + ibuprofen 10 mg/kg single dose of each	Primary 1. Reduction in temperature at h 4 Secondary 1. Percent reduction of temperature at h 4 2. Proportion of afebrile children at 1, 2, 3, and 4 h 3. Adverse drug events occurring in the 4 h period

*ED, emergency department, NCCPC, non-communicating children's pain checklist*.

### Risk of Bias Assessment

**Figure 2:**
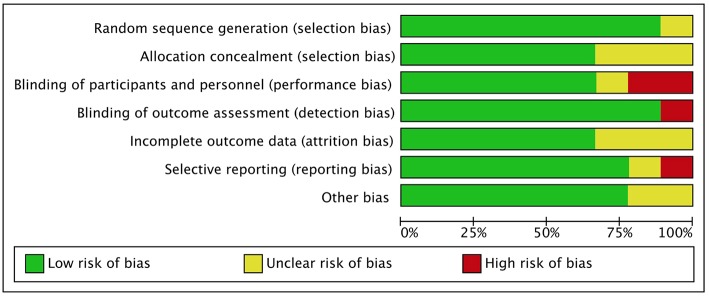
Risk of bias graph.

**Figure 3:**
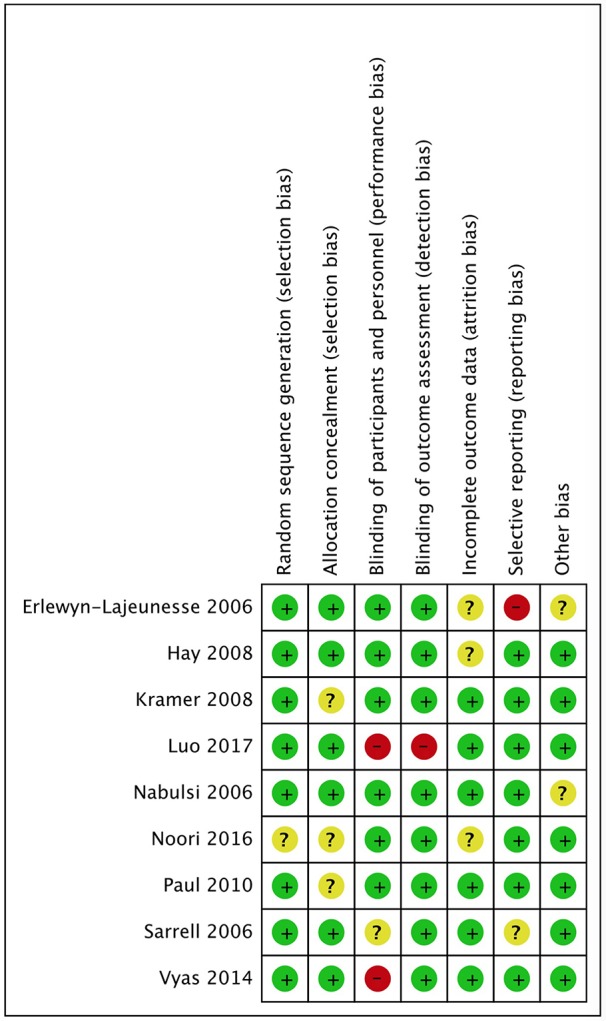
Risk of bias summary.

The results and summary of risk of bias assessment for included studies are shown in Figures 2, 3. The majority of included studies presented low risk of bias. Performance bias (blinding of participants and personnel) was the dominant cause of high risk of bias, with two studies (36, 41) presenting high risk, because not all the investigators were blinded to the intervention, and one study (46) unclear risk of bias. One study had a high risk of bias regarding blinding of outcome assessment because all investigators were un-blinded (41). Selection bias was high in one study (34), because the authors decided not to report data on mean temperature at hour 2 and on time spent on the unit.

### Effects of Interventions

Analyses were performed for the following comparisons: alternating therapy vs. single agent, combined therapy vs. single agent and combined vs. alternating therapy (Tables 3, 4). We decided to present only the analyses that included additional studies, compared to the analyses reported in the previous Cochrane review (22).

**Table 3:** Comparison 1: combined therapy vs. single agent.

**Outcome or subgroup**	**Studies**	**Participants**	**Statistical method**	**Effect estimate**
Proportion remaining febrile	3		RR (M-H, Random, 95% CI)	Subtotals only
Hour 1	2	133	RR (M-H, Random, 95% CI)	0.46 [0.20, 1.07]
Hour 4	3	289	RR (M-H, Random, 95% CI)	0.18 [0.06, 0.53]
Hour 6	1	40	RR (M-H, Random, 95% CI)	0.10 [0.01, 0.71]
Mean temperature (°C)	4		MD (IV, Random, 95% CI)	Subtotals only
Hour 1	2	163	MD (IV, Random, 95% CI)	−0.29 [−0.45, −0.13]
Hour 4	3	713	MD (IV, Random, 95% CI)	−0.12 [−0.34, 0.10]
Hour 6	2	580	MD (IV, Random, 95% CI)	−0.04 [−0.13, 0.05]

*RR, risk ratio; MD, mean difference*.

**Table 4:** Comparison 2: alternating therapy vs. single agent.

**Outcome or subgroup**	**Studies**	**Participants**	**Statistical method**	**Effect estimate**
Proportion remaining febrile	3		RR (M-H, Random, 95% CI)	Subtotals only
Hour 4	2	511	RR (M-H, Random, 95% CI)	0.33 [0.07, 1.43]
Hour 6	3	580	RR (M-H, Random, 95% CI)	0.30 [0.15, 0.57]
NCCPC score	2		MD (IV, Random, 95% CI)	−2.57 [−3.36, −1.79]
Day 1	2	935	MD (IV, Random, 95% CI)	−1.32 [−2.47, −0.17]
Day 2	1	464	MD (IV, Random, 95% CI)	−3.76 [−4.18, −3.34]
Day 3	1	464	MD (IV, Random, 95% CI)	−3.64 [−4.08, −3.20]
Doses of medication per child	2		MD (IV, Random, 95% CI)	−0.92 [−1.36, −0.48]
Day 1	2	935	MD (IV, Random, 95% CI)	−0.44 [−1.34, 0.47]
Day 2	1	464	MD (IV, Random, 95% CI)	−1.39 [−2.29, −0.49]
Day 3	1	464	MD (IV, Random, 95% CI)	−1.38 [−1.49, −1.28]

*RR, risk ratio; MD, mean difference; NCCPC, non-communicating children's pain checklist*.

#### Combined Therapy vs. Single Agent

Five studies (34, 36, 42–44) compared therapy with paracetamol and ibuprofen together at baseline and single agent therapy. Outcome measures were: proportion of children remaining febrile and mean temperature at one, 4 and 6 h from baseline.

**Figure 4:**
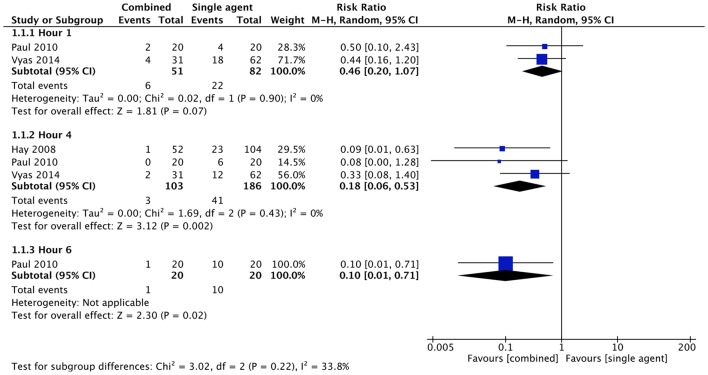
Comparison: combined vs. single agent, Outcome: proportion remaining febrile.

No statistical difference was found in the proportion of children remaining febrile at 1 h after the initial administration of therapy (RR 0.46, with 95% CI 0.20–1.07, 133 participants, two trials). Proportion of children remaining febrile after 4 and 6 h from baseline was lower in the combined therapy groups. RR was 0.18 at 4 h (with 95% CI 0.06–0.53, 289 participants, three trials) and 0.10 at 6 h (with 95% CI 0.01–0.71, 40 participants, one trial) (Figure 4).

**Figure 5:**
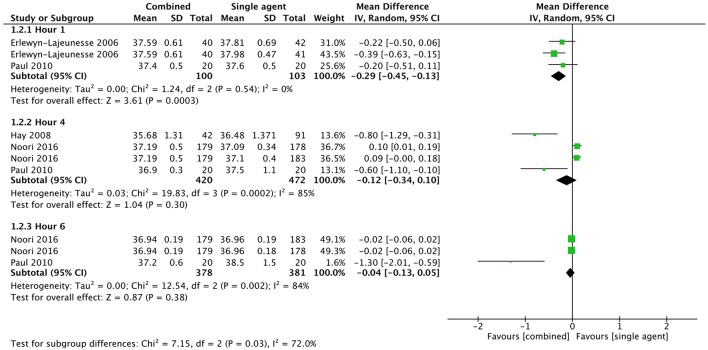
Comparison: combined vs. single agent, Outcome: mean temperature.

Mean temperature was lower in the combined therapy group at 1 h after the initial administration of therapy (MD −0.29, CI −0.45 to −0.13, 163 participants, two trials). No statistical difference was found in mean temperature at 4 h (MD −0.12, CI −0.34 to 0.10, 713 participants, three trials) and 6 h (MD −0.04, CI −0.13 to 0.05, 580 participants, two trials) from baseline (Figure 5).

#### Alternating Therapy vs. Single Agent

Five studies (41, 44–47) compared alternating therapy with paracetamol and ibuprofen and single agent therapy. Outcome measures were: proportion of children remaining febrile at 4 and 6 h from baseline, NCCPC score and doses of medication per child at one, 2 and 3 days from baseline.

**Figure 6:**
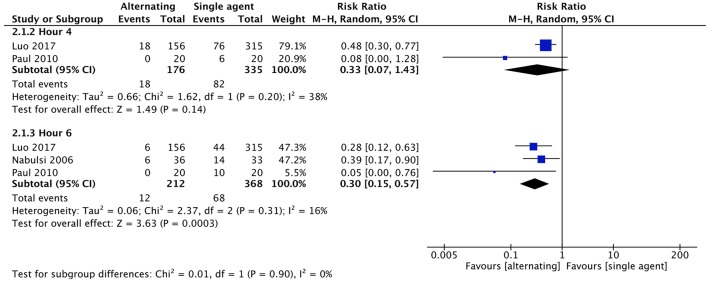
Comparison: alternating vs. single agent, Outcome: proportion remaining febrile.

No statistical difference was found in the proportion of children remaining febrile at 4 h after the initial administration of therapy (RR 0.33 with 95% CI 0.07–1.43, 511 participants, two trials). Proportion of children remaining febrile after 6 h from baseline was lower in the alternating therapy group (RR 0.30 with 95% CI 0.15–0.57, 580 participants, three trials) (Figure 6).

**Figure 7:**
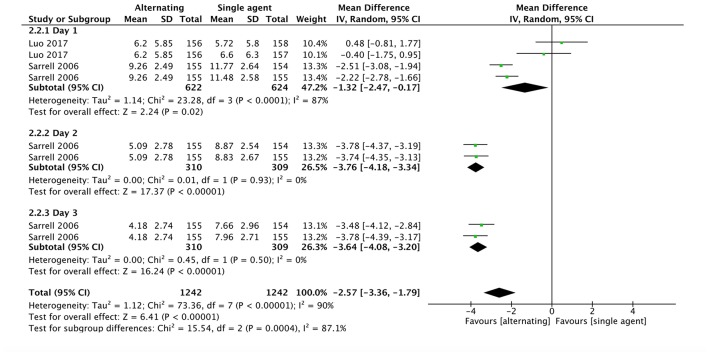
Comparison: alternating vs. single agent, Outcome: NCCPC score.

Two studies (41, 46) evaluated NCCPC scores as measure of child discomfort. Even though Luo et al. (41) found no significant differences in mean NCCPC scores across groups, pooled data at day one revealed a statistically significant difference between alternating and single agent groups, with NCCPC scores being lower in children receiving alternating therapy than in children receiving either of the single agents (MD −1.32, 95% CI −2.47 to −0.17, 935 participants, two trials). According to Sarrell et al. (46), in the alternating therapy group NCCPC scores were lower also on days 2 (MD −3.76, 95% CI −4.18 to −3.34, 464 participants, one trial) and 3 (MD −3.64, 95% CI −4.08 to −3.20, 464 participants, one trial) (Figure 7).

**Figure 8:**
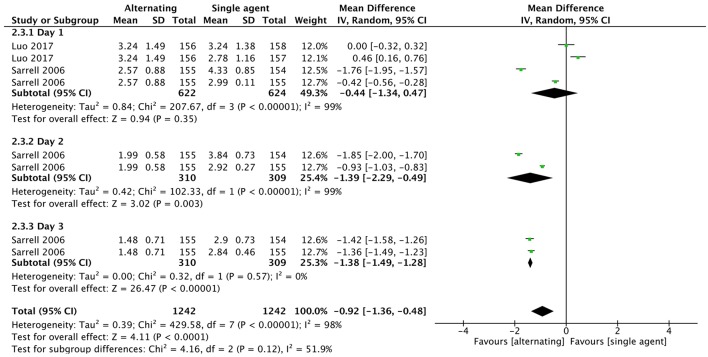
Comparison: alternating vs. single agent, Outcome: doses of medication per child.

The same two studies (41, 46) evaluated the mean number of doses of antipyretic per child. No statistical difference was found between groups in doses of antipyretic per child during the first 24 h (MD −0.44, 95% CI −1.34 to 0.47, 935 participants, two trials). Sarrell et al. (46) showed that children in the alternating group required fewer doses of antipyretic during day 2 (MD −1.39, 95% CI −2.29 to −0.49, 464 participants, one trial) and day 3 (MD −1.38, 95% CI −1.49 to −1.28, 464 participants, one trial) (Figure 8).

#### Adverse Events

Overall, no serious adverse events were observed in any studies. The reported non-severe adverse events were gastrointestinal symptoms (mainly nausea, abdominal pain, diarrhea, and vomiting) (36, 41, 43, 47), mild elevated liver enzymes and mild abnormal renal function, normalized by 14 day follow-up (46), skin rash (36, 41, 43), asthma (41). Those events were distributed similarly between the different comparison groups (Table 5).

**Table 5:** Adverse effects.

**Studies**	***N***	**Duration of follow-up**	**Serious adverse events**	**Non-serious adverse events**
Erlewyn-Lajeunesse et al. (34)	123	2 h	Not reported	Not reported
Hay et al. (43)	156	5 days	5 admission to hospital, reasons not reported; no difference between groups	Paracetamol group: diarrhea, *n* = 10, vomiting, *n* = 6, rash, *n* = 2, cough, *n* = 2; ibuprofen group: diarrhea, *n* = 9, vomiting, *n* = 3, rash, *n* = 2; combination group: diarrhea, *n* = 12, vomiting, *n* = 2, rash, *n* = 1, cough, *n* = 1
Kramer et al. (47)	38	6 h	None observed	Diarrhea, flatulence, emesis, decreased appetite, epigastric pain, nausea, headache, insomnia; *n* = 8, no difference between groups
Luo et al. (41)	474	24 h	None observed	Paracetamol group: gastrointestinal symptoms, *n* = 133, rash, *n* = 14; ibuprofen group: gastrointestinal symptoms n = 134, rash, *n* = 13, asthma, *n* = 2; alternating group: gastrointestinal symptoms, *n* = 132, rash, *n* = 13
Nabulsi et al. (45)	70	8 h	None observed	None observed
Noori et al. (42)	540	6 h	None observed	None observed
Paul et al. (44)	46	6 h	Not reported	Not reported
Sarrell et al. (46)	480	14 days	None observed	Paracetamol group: mild elevated liver enzymes, *n* = 4, mild abnormal renal function, *n* = 5; ibuprofen group: mild elevated liver enzymes, *n* = 2, mild abnormal renal function, *n* = 4; alternating group: mild elevated liver enzymes, *n* = 2, mild abnormal renal function, *n* = 5; all normalized by 14 day follow up
Vyas et al. (36)	99	4 h	None observed	Paracetamol group: vomiting, *n* = 1, abdominal pain, *n* = 1, (doubtful relationship to treatment); ibuprofen group: nausea, *n* = 1, abdominal pain, *n* = 1, maculopapular skin rash, *n* = 1, (possible relationship to treatment); combination group: vomiting, *n* = 1 (doubtful relationship to treatment), abdominal pain, *n* = 2, skin rash, *n* = 1 (possible relationship to treatment)

### Quality of the Evidence

The quality of the evidence was low or very low for all outcomes except for two subgroups in the comparison combined therapy vs. single agent (proportion remaining febrile at 4 h, mean temperature at 1 h), which did not suffer of significant inconsistency or imprecision (Tables 6, 7; Supplementary Tables 1, 2).

**Table 6:** Summary of findings: combined therapy vs. single agent.

**Outcomes**	Anticipated absolute effects^*^ **(95% CI)**		**Relative effect (95% CI)**	**N of participants (studies)**	**Certainty of the evidence (GRADE)**
	**Risk with single agent**	**Risk with combined**			
Proportion remaining febrile—Hour 1	Study population		RR 0.46 (0.20 to 1.07)	133 (2 RCTs)	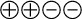 LOW
	268 per 1.000	123 per 1.000 (54 to 287)			
Proportion remaining febrile—Hour 4	Study population		RR 0.18 (0.06 to 0.53)	289 (3 RCTs)	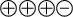 MODERATE
	220 per 1.000	40 per 1.000 (13 to 117)			
Proportion remaining febrile—Hour 6	Study population		RR 0.10 (0.01 to 0.71)	40 (1 RCT)	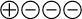 VERY LOW
	500 per 1.000	50 per 1.000 (5 to 355)			
Mean temperature (°C)—Hour 1	The mean temperature (°C)—Hour 1 ranged from 37.6 to 37.98	MD 0.29 lower (0.45 lower to 0.13 lower)	-	203 (2 RCTs)	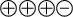 MODERATE
Mean temperature (°C)—Hour 4	The mean temperature (°C)—Hour 4 ranged from 36.48 to 37.5	MD 0.12 lower (0.34 lower to 0.1 higher)	-	892 (3 RCTs)	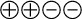 LOW
Mean temperature (°C)—Hour 6	The mean temperature (°C)—Hour 6 ranged from 36.96 to 38.5	MD 0.04 lower (0.13 lower to 0.05 higher)	-	759 (2 RCTs)	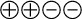 LOW

* *The risk in the intervention group (and its 95% confidence interval) is based on the assumed risk in the comparison group and the relative effect of the intervention (and its 95% CI). CI, Confidence interval; RR, Risk ratio; OR, Odds ratio; RCT Randomized Controlled Trial*.

**Table 7:** Summary of findings: alternating therapy vs. single agent.

**Outcomes**	Anticipated absolute effects^*^ **(95% CI)**		**Relative effect (95% CI)**	**N of participants (studies)**	**Certainty of the evidence (GRADE)**
	**Risk with single agent**	**Risk with alternating**			
Proportion remaining febrile—Hour 4	Study population		RR 0.33 (0.07 to 1.43)	511 (2 RCTs)	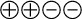 LOW
	245 per 1.000	81 per 1.000 (17 to 350)			
Proportion remaining febrile—Hour 6	Study population		RR 0.30 (0.15 to 0.57)	580 (3 RCTs)	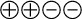 LOW
	185 per 1.000	55 per 1.000 (28 to 105)			
NCCPC score—Day 1	NCCPC score—Day 1 ranged from 5.72 to 11.77	MD 1.32 lower (2.47 lower to 0.17 lower)	-	1246 (2 RCTs)	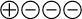 VERY LOW
NCCPC score—Day 2	NCCPC score—Day 2 ranged from 8.83 to 8.87	MD 3.76 lower (4.18 lower to 3.34 lower)	-	619 (1 RCT)	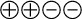 LOW
NCCPC score—Day 3	NCCPC score—Day 3 ranged from 7.66 to 7.96	MD 3.64 lower (4.08 lower to 3.2 lower)	-	619 (1 RCT)	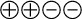 LOW
Doses of medication per child—Day 1	The mean doses of medication per child—Day 1 ranged from 2.78 to 4.33	MD 0.44 lower (1.34 lower to 0.47 higher)	-	1246 (2 RCTs)	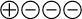 VERY LOW
Doses of medication per child—Day 2	The mean doses of medication per child—Day 2 ranged from 2.92 to 3.84	MD 1.39 lower (2.29 lower to 0.49 lower)	-	619 (1 RCT)	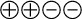 LOW
Doses of medication per child—Day 3	The mean doses of medication per child—Day 3 ranged from 2.84 to 2.9	MD 1.38 lower (1.49 lower to 1.28 lower)	-	619 (1 RCT)	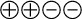 LOW

* *The risk in the intervention group (and its 95% confidence interval) is based on the assumed risk in the comparison group and the relative effect of the intervention (and its 95% CI). CI, Confidence interval; RR, Risk ratio; OR. Odds ratio; RCT, Randomized Controlled Trial*.

## Discussion

In the present study we updated literature findings regarding the efficacy and safety of combined/alternating use of antipyretics in children, 5 years after the publication of the previous Cochrane review available at this regard (22). Several new additional studies were available, including one publication exploring the effect on the child's discomfort, which represents the main goal of antipyretic therapy in children. Even if, overall, the results of our systematic review are aligned with the ones presented in the previous Cochrane review (22), including the additional studies, some differences were observed. The main discrepancy concerns the effect of combined therapy on the mean temperature: while administering combined paracetamol and ibuprofen therapy to febrile children resulted in a lower mean temperature at 1 h after treatment, the mean difference in temperature at 4 and 6 h was no more statistically significant. Moreover, the reduction in body temperature reached after 1 h was small (0.29°C). Although there is no international consensus on the definition of a clinically relevant reduction in temperature, most authors considered meaningful only reductions higher than 1°C. Thus, a reduction in temperature of 0.29°C can be considered as not clinically relevant.

There is, instead, a significant difference in the proportion of children reaching apyrexia at 4 and 6 h after combined treatment and at 6 h after alternating treatment, compared to children treated with monotherapy, consistently to previous findings (19). These results support the evidence that giving both paracetamol and ibuprofen, in a combined or alternating regimen, could be more effective in treating fever, compared to monotherapy alone. Nevertheless, the confidence interval of pooled results was wide, with a superior limit close to one, suggesting a possible imprecision of the point estimates.

Regarding the NCCPC score, in this review the overall mean difference among groups was lower than the one reported in the 2013 Cochrane review, which was based on a single study. According to Luo et al. (41), indeed, there was no improvement on the child's discomfort during the first 24 h with alternating treatment vs. monotherapy. Moreover, the pooled mean difference in the number of medications per child used during the first 24 h was not significantly different.

During the follow-up period, no serious adverse events were observed. Furthermore, non-severe adverse events were spread similarly between groups. However, this study has not the statistical power to detect rare adverse drug reactions. Additional studies are needed to confirm the safety of combined or alternating therapy. Indeed, some studies revealed warning signs regarding the safety of combined therapy, focusing on possible renal injury (48, 49).

Finally, some authors underlined that the risk of administration errors by the caregivers could be increased using combined or alternating therapy (11).

### Limitations of the Study

This study shows some limitations. There was significant heterogeneity between studies, both in the population sample (age, etiology of fever, comorbidities), both in the methods used (doses of medications, regimens and frequency of administration, methods of measuring body temperature). Due to the small number of trials, we could not quantify the impact of these variations. Moreover, the small number of trials did not allow us to build funnel plots and we could not exclude the possibility of publication bias. Finally, the quality of the evidence was generally poor.

## Conclusion

According to results from our updated review, combined or alternating therapy resulted more effective than monotherapy in reducing body temperature. However, the benefit seems slighter than those reported in the previous 2013 Cochrane review (22), and probably not clinically relevant. Similar results were observed by pooling data from two studies evaluating the effect on the child's discomfort and number of doses of medication. To date, evidences are not enough to encourage combined or alternating paracetamol and ibuprofen instead of monotherapy to treat febrile children. Our updated meta-analysis results reinforce the current recommendations of most of the international guidelines, discouraging this prescription habit.

Further studies are needed, particularly RCT on a large sample of febrile children randomized into treatment groups (combined/alternated vs. single agent), in order to reach a clearer estimate of effects, and large post-marketing surveillance studies, in order to detect any rare severe adverse drugs reaction.

## Author Contributions

EC conceptualized the study. GT and EC retrieved the pertinent literature, reviewed and revised the manuscript and take responsibility for the paper as a whole. GT and MC carried out the analyses and drafted the initial manuscript. EC supervised the analyses. MdM supervised the project and gave conceptual advice. All authors approved the final manuscript as submitted.

### Conflict of Interest Statement

The authors declare that the research was conducted in the absence of any commercial or financial relationships that could be construed as a potential conflict of interest.
