# Supplementary material for: Prescribing Controversies: An Updated Review and Meta-Analysis on Combined/Alternating Use of Ibuprofen and Paracetamol in Febrile Children
Source: Front Pediatr. 2019 Jun 5;7:217. doi: 10.3389/fped.2019.00217 (PMC6560148; doi:10.3389/fped.2019.00217)
Supplement: Supplementary file 1 [file Table_1.DOCX]

**Supplementary Table 1**

**Quality of the evidence assessment**

Combined therapy compared to single agent therapy for febrile children

| **Certainty assessment** | | | | | | | **№ of patients** | | **Effect** | | **Certainty** | **Importance** |
| --- | --- | --- | --- | --- | --- | --- | --- | --- | --- | --- | --- | --- |
| **№ of studies** | **Study design** | **Risk of bias** | **Inconsistency** | **Indirectness** | **Imprecision** | **Other considerations** | **Combined** | **single agent** | **Relative (95% CI)** | **Absolute (95% CI)** |  |  |
| Proportion remaining febrile - Hour 1 | | | | | | | | | | | | |
| 2 | randomised trials | serious | not serious | not serious | serious | publication bias strongly suspected all plausible residual confounding would reduce the demonstrated effect | 6/51 (11.8%) | 22/82 (26.8%) | **RR 0.46** (0.20 to 1.07) | **145 fewer per 1.000** (from 19 more to 215 fewer) | ⨁⨁◯◯ LOW |  |
| Proportion remaining febrile - Hour 4 | | | | | | | | | | | | |
| 3 | randomised trials | serious | not serious | not serious | not serious | publication bias strongly suspected all plausible residual confounding would reduce the demonstrated effect | 3/103 (2.9%) | 41/186 (22.0%) | **RR 0.18** (0.06 to 0.53) | **181 fewer per 1.000** (from 104 fewer to 207 fewer) | ⨁⨁⨁◯ MODERATE |  |
| Proportion remaining febrile - Hour 6 | | | | | | | | | | | | |
| 1 | randomised trials | serious | not serious | not serious | very serious | publication bias strongly suspected all plausible residual confounding would reduce the demonstrated effect | 1/20 (5.0%) | 10/20 (50.0%) | **RR 0.10** (0.01 to 0.71) | **450 fewer per 1.000** (from 145 fewer to 495 fewer) | ⨁◯◯◯ VERY LOW |  |
| Mean temperature (°C) - Hour 1 | | | | | | | | | | | | |
| 2 | randomised trials | serious | not serious | not serious | not serious | publication bias strongly suspected all plausible residual confounding would reduce the demonstrated effect | 100 | 103 | - | MD **0.29 lower** (0.45 lower to 0.13 lower) | ⨁⨁⨁◯ MODERATE |  |
| Mean temperature (°C) - Hour 4 | | | | | | | | | | | | |
| 3 | randomised trials | serious | serious | not serious | not serious | publication bias strongly suspected all plausible residual confounding would reduce the demonstrated effect | 420 | 472 | - | MD **0.12 lower** (0.34 lower to 0.1 higher) | ⨁⨁◯◯ LOW |  |
| Mean temperature (°C) - Hour 6 | | | | | | | | | | | | |
| 2 | randomised trials | serious | serious | not serious | not serious | publication bias strongly suspected all plausible residual confounding would reduce the demonstrated effect | 378 | 381 | - | MD **0.04 lower** (0.13 lower to 0.05 higher) | ⨁⨁◯◯ LOW |  |

**CI:** Confidence interval; **RR:** Risk ratio; **MD:** Mean difference
