# Supplementary material for: Prescribing Controversies: An Updated Review and Meta-Analysis on Combined/Alternating Use of Ibuprofen and Paracetamol in Febrile Children
Source: Front Pediatr. 2019 Jun 5;7:217. doi: 10.3389/fped.2019.00217 (PMC6560148; doi:10.3389/fped.2019.00217)
Supplement: Supplementary file 2 [file Table_2.DOCX]

**Supplementary Table 2**

**Quality of the evidence assessment**

Alternating therapy compared to single agent therapy for febrile children

| **Certainty assessment** | | | | | | | **№ of patients** | | **Effect** | | **Certainty** | **Importance** |
| --- | --- | --- | --- | --- | --- | --- | --- | --- | --- | --- | --- | --- |
| **№ of studies** | **Study design** | **Risk of bias** | **Inconsistency** | **Indirectness** | **Imprecision** | **Other considerations** | **Alternating** | **single agent** | **Relative (95% CI)** | **Absolute (95% CI)** |  |  |
| Proportion remaining febrile - Hour 4 | | | | | | | | | | | | |
| 2 | randomised trials | serious | serious | not serious | not serious | publication bias strongly suspected all plausible residual confounding would reduce the demonstrated effect | 18/176 (10.2%) | 82/335 (24.5%) | **RR 0.33** (0.07 to 1.43) | **164 fewer per 1.000** (from 105 more to 228 fewer) | ⨁⨁◯◯ LOW |  |
| Proportion remaining febrile - Hour 6 | | | | | | | | | | | | |
| 3 | randomised trials | serious | serious | not serious | not serious | publication bias strongly suspected all plausible residual confounding would reduce the demonstrated effect | 12/212 (5.7%) | 68/368 (18.5%) | **RR 0.30** (0.15 to 0.57) | **129 fewer per 1.000** (from 79 fewer to 157 fewer) | ⨁⨁◯◯ LOW |  |
| Non-communicating Children’sPain Checklist (NCCPC) score - Day 1 | | | | | | | | | | | | |
| 2 | randomised trials | very serious | very serious | not serious | not serious | publication bias strongly suspected all plausible residual confounding would reduce the demonstrated effect | 622 | 624 | - | MD **1.32 lower** (2.47 lower to 0.17 lower) | ⨁◯◯◯ VERY LOW |  |
| Non-communicating Children’sPain Checklist (NCCPC) score - Day 2 | | | | | | | | | | | | |
| 1 | randomised trials | very serious | not serious | not serious | not serious | publication bias strongly suspected all plausible residual confounding would reduce the demonstrated effect | 310 | 309 | - | MD **3.76 lower** (4.18 lower to 3.34 lower) | ⨁⨁◯◯ LOW |  |
| Non-communicating Children’sPain Checklist (NCCPC) score - Day 3 | | | | | | | | | | | | |
| 1 | randomised trials | very serious | not serious | not serious | not serious | publication bias strongly suspected all plausible residual confounding would reduce the demonstrated effect | 310 | 309 | - | MD **3.64 lower** (4.08 lower to 3.2 lower) | ⨁⨁◯◯ LOW |  |
| Doses of medication per child - Day 1 | | | | | | | | | | | | |
| 2 | randomised trials | serious | very serious | not serious | not serious | publication bias strongly suspected all plausible residual confounding would reduce the demonstrated effect | 622 | 624 | - | MD **0.44 lower** (1.34 lower to 0.47 higher) | ⨁◯◯◯ VERY LOW |  |
| Doses of medication per child - Day 2 | | | | | | | | | | | | |
| 1 | randomised trials | very serious | not serious | not serious | not serious | publication bias strongly suspected all plausible residual confounding would reduce the demonstrated effect | 310 | 309 | - | MD **1.39 lower** (2.29 lower to 0.49 lower) | ⨁⨁◯◯ LOW |  |
| Doses of medication per child - Day 3 | | | | | | | | | | | | |
| 1 | randomised trials | very serious | not serious | not serious | not serious | publication bias strongly suspected all plausible residual confounding would reduce the demonstrated effect | 310 | 309 | - | MD **1.38 lower** (1.49 lower to 1.28 lower) | ⨁⨁◯◯ LOW |  |

**CI:** Confidence interval; **RR:** Risk ratio; **MD:** Mean difference
